# Antioxidative Peptides Derived from Enzyme Hydrolysis of Bone Collagen after Microwave Assisted Acid Pre-Treatment and Nitrogen Protection

**DOI:** 10.3390/ijms11114297

**Published:** 2010-11-01

**Authors:** Yun-Jian Lin, Guo-Wei Le, Jie-Yun Wang, Ya-Xin Li, Yong-Hui Shi, Jin Sun

**Affiliations:** 1 School of Food Science and Technology, Jiangnan University, Wuxi 214122, China; E-Mails: yunjianl@163.com (Y.-J.L.); wang_jieyun@yahoo.com.cn (J.-Y.W.); liyaxin1220@163.com (Y.-X.L.); 2 State Key Laboratory of Food Science and Technology, Jiangnan University, Wuxi 214122, China; E-Mails: yhshi@jiangnan.edu.cn (Y.-H.S.); sunj@jiangnan.edu.cn (J.S.)

**Keywords:** bone collagen, nitrogen protection, microwave assisted acid pre-treatment, enzyme hydrolysis, antioxidant activity

## Abstract

This study focused on the preparation method of antioxidant peptides by enzymatic hydrolysis of bone collagen after microwave assisted acid pre-treatment and nitrogen protection. Phosphoric acid showed the highest ability of hydrolysis among the four other acids tested (hydrochloric acid, sulfuric acid and/or citric acid). The highest degree of hydrolysis (DH) was 9.5% using 4 mol/L phosphoric acid with a ratio of 1:6 under a microwave intensity of 510 W for 240 s. Neutral proteinase gave higher DH among the four protease tested (Acid protease, neutral protease, Alcalase and papain), with an optimum condition of: (1) ratio of enzyme and substrate, 4760 U/g; (2) concentration of substrate, 4%; (3) reaction temperature, 55 °C and (4) pH 7.0. At 4 h, DH increased significantly (*P* < 0.01) under nitrogen protection compared with normal microwave assisted acid pre-treatment hydrolysis conditions. The antioxidant ability of the hydrolysate increased and reached its maximum value at 3 h; however DH decreased dramatically after 3 h. Microwave assisted acid pre-treatment and nitrogen protection could be a quick preparatory method for hydrolyzing bone collagen.

## 1. Introduction

Collagen is one of the longest fibrous structural proteins. Collage is an extracellular matrix protein, which plays an important role in many animal tissues within the skeletal, muscular and cardiovascular network tissues [1]. Native collagen is very hard to digest; their functions are quite different from those of globular proteins such as enzymes. Tough bundles of collagen called collagen fibers are a major component of the extracellular matrix that supports most tissues and gives cells structure from the outside, but collagen is also found inside certain cells. Collagen has great tensile strength, and is the main component of fascia, cartilage, ligaments, tendons, bone and skin [1,2]. As raw materials in medicine and food industries, collagen peptides are used as important active components because of their excellent bioactivity, good biocompatibility, good penetrability and non-irritation of the body [3]. Modification of a protein is usually realized by physical, chemical, or enzymatic treatments, which change its structure and consequently its physicochemical and bioactive properties [4]. Hydrolyzed collagens from fish, porcine and bovine origin are now used in such consumables as functional food, beverages and dietary supplements. Many reports indicated that collagen peptides may act as a messenger, and trigger the synthesis reorganization of new collagen fibers by stimulating fibroblast cells [3,5]. Recently, some studies showed that hydrolyzed collagen increases the fibroblast density and the diameter of collagen fibrils in the dermis [6]. Thus, hydrolyzed collagen may improve the mechanical strength of the skin by increasing decorin ratio [7].

Many human diseases are known to be caused by free radicals and the natural antioxidants that can act as free radical scavengers. Protein hydrolysates with antioxidant properties, in particular, have become a topic of great interest for the pharmaceutical, health food, as well as the food processing and preservation industries [8,9].

Bioactive peptides commonly contain 3–20 amino acids per peptide as inactive sequences within large proteins and are released when the parent protein is hydrolyzed by digestive enzymes (*in vitro* and *in vivo*), by microbial enzymes, or during food processing [10,11]. Enzymatic hydrolysis of proteins is one approach used to release bioactive peptides and is widely applied to improve functional and nutritional properties of protein sources [12]. Collagen is known to be rich in hydrophobic amino acids. Therefore, collagen is expected to provide natural antioxidant peptides and exert higher antioxidant properties [13,14].

A previous study of Lloyd [15] demonstrated that most nonspecific proteolytic enzymes could not cleave the collagen fibers from their sides, and their action on collagen fibers is also limited to cut the ends part. A series of studies has recently demonstrated that the use of microwave radiation for sample hydrolysis is one of the most important developments of protein identification [16]. Microwave assisted digestion of protein by acid hydrolysis results in reduction of the overall hydrolysis time from several hours to a few minutes [17–20]. This method might be useful for mobilizing the triple-helical part, which might hasten the enzyme hydrolysis of collagen.

Pro, which is rich in collagen, is prone to oxidation where the hydrogen atom is abstracted from OH- and N-containing group [21]. The oxidation of the side chains of proline have been shown to yield carbonyl derivatives [22], which might cause impairment to antioxidant activity of collagen peptide. Thus, dissolved oxygen under agitation might be an inhibitor for producing antioxidant peptides from hydrolysis of collagen.

The bioactive molecules in the bone collagen hydrolysate responsible for antioxidant properties are peptides that are released upon hydrolysis [23]. As a result, the objectives of this study were to: develop the method of preparation of antioxidant peptides by enzyme hydrolysis of bone collagen after microwave assisted acid pre-treatment under nitrogen protection; to study the effect of removing oxygen during the time of hydrolysis; and investigate the hydrolysate antioxidant activity.

## 2. Results and Discussions

### 2.1. Effect of Microwave Assisted Acid Pre-Treatment on Bone Collagen

Microwave is a high frequency electromagnetic wave. It is through the polarization of molecules, rather than molecular collisions, that energy is transferred. The polarized degree of the dipole is dependent on the dipolar characteristic of molecules. The rapid absorption of microwave energy significantly shortened protein hydrolysis time due to the strong polarization of water molecules. Compared with other heating reaction systems, the microwave assisted acid hydrolysis system provides more active centers. The energy transmits to all active centers of reaction at the same time, resulting in an instantaneous hydrolysis, which substantially increases hydrolysis efficiency.

Traditionally, collagen was hydrolyzed by chemical and enzymatic methods. Acids and alkalis cut the protein into smaller molecules. However, molecular distribution of hydrolysate is uneven and accompanied with a longer hydrolysis reaction time. Zhong [23] investigated microwave assisted acid hydrolysis of cytochrome C, phagocytic opsonic and other proteins; it was found that conventional heating hydrolysis methods lead to protein aggregation and obstruct further hydrolysis, while microwave radiation prevents protein aggregation and denaturation, and proved helpful for hydrolyzing both hydrophilic and hydrophobic proteins.

In the present study, phosphoric acid showed the highest ability to hydrolyze collagen through microwave assisted hydrolysis among the four tested acids (Table 1). Furthermore, phosphoric acid showed higher ABTS^+^ cleaving capacity than the other acids used, in addition, sulfuric acid could present harmful effects on the body which may disqualify it from food application (Table 1). The hydrolysis condition was further optimized. The highest DH (9.5%) was obtained with 4 mol/L phosphoric acid used for liquid and material with ratio 6:1 under microwave intensity of 510 W for 240 s.

Collagen hydrolysis is a process in which peptide bonds are damaged and broken, generating the final formation of peptides. During the microwave assisted acid hydrolysis process, the acid can attack the peptide bond. The natural frequency of protein (5 × 1010 Hz) is just at the microwave frequencies range of action (3× 108∼3 × 1011 Hz), so that protein molecules vibrate alternately in the electromagnetic field and the positive and negative charges of each molecules are affected by alternating electromagnetic power. This results in the degradation and spatial structural change of the protein and improves the acid's effect and increases the efficiency of the enzyme by adding a target site and shortens the hydrolysis time significantly.

### 2.2. Effect of Microwave Assisted Acid Pre-Treatment on Enzyme Digestion of Bone Collagen

**Figure 1.**
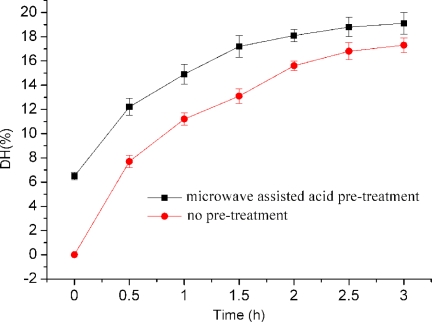
Degree of hydrolysis with microwave acid-pretreatment and without (non-pretreated), with no nitrogen protection of bone collagen, using Neutral proteinase, at the indicated times. Results are presented as the means (n = 3) ± SD. The data mean is significantly different at (*P* < 0.05).

Four types of enzymes (acid protease, neutral protease, alcalase and papain) were tested for their ability to hydrolyze swine bone collagen. It is shown in Figure 1 that higher DH was produced when neutral protease was used without nitrogen protection. The condition was further optimized for neutral proteinase to hydrolyze swine bone collagen. The best conditions were found to be: (1) enzyme and substrate ratio, 4760 U/g; (2) concentration of substrate, 4%; (3) reaction temperature, 55 °C and (4) pH, 7.0.

**Figure 2.**
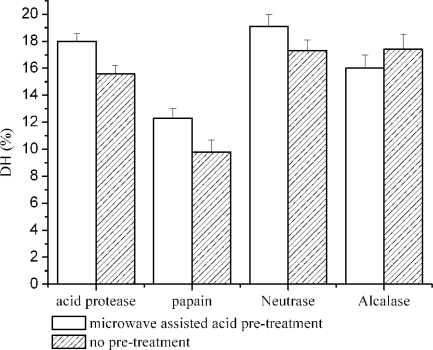
Effect of microwave assisted acid pre-treatment on degree of hydrolysis of collagen by different protease (acid protease, neutrase, papain and alcalase) at time =3 h. Results are presented as the means (n = 3) ± SD. The data mean is significantly different at (*P* < 0.05).

The hydrolysis was done using untreated and acid pretreated swine bone collagen. It is shown in Figure 1 that microwave assisted acid pre-treatment increased DH up to 2 h; thereafter slight changes occurred on hydrolysis. Highest DH (19.1 ± 0.9) was obtained at 3 h. The effect of microwave assisted acid pre-treatment was further studied on hydrolysis efficiency of other enzymes. Figure 2 shows that pretreatment significantly improved (*P* < 0.05) the efficiency of enzymes; however no effect was shown on the activity of alcalase.

Enzymatic hydrolysis is an effective method for preparing bioactive peptides, such as the antihypertensive peptides, antioxidant peptides, immune peptides, *etc*. Lin [18] has systematically studied hydrolysis of different proteins (myoglobin, cytochrome, lysozyme, *etc*.) in different organic solvent microwave-assisted enzyme-catalyzed reactions. He found that trypsin activity did not increase with different solvents; rather, he still considered this method better than the conventional methods. In the conventional method, enzyme inactivation occurs due to slow warming speed, while hydrolysis completes prior to the complete inactivation of the enzyme in microwave heating methods; this corroborated investigations of Juan [19] and Vesper [20].

**Figure 3.**
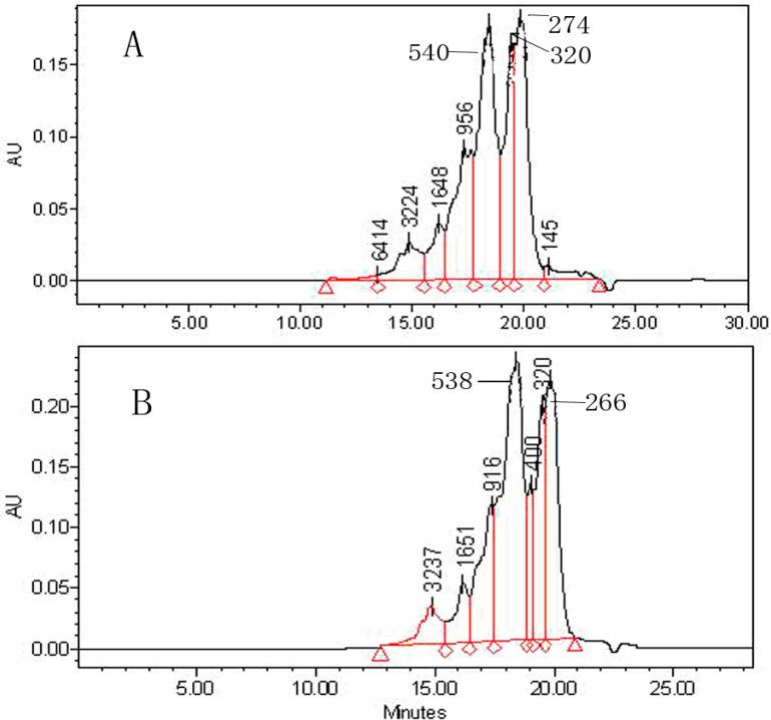
The molecular weight distributions of collagen hydrolysate (using neutrase) microwave assisted acid pre-treatment: (**A**) Enzyme treatment for 3 h for native collagen and (**B**) enzyme treatment for 1.5 h microwave assisted acid pre-treated collagen.

Figure 3 shows molecular weight (MW) distributions of 3 h treatment swine bone collagen hydrolysate by neutrase. Approximate 82% of MW distributions were found in the range of <1000 Da (Figure 3A), indicating that hydrolysate contained a large proportion of low-molecular-weight peptides with 2–6 amino acidresidues. The use of microwave assisted acid pre-treatment shortens the time of hydrolysis to 1.5 h and products of low molecular weight were achieved (80% MW are <1000 Da) (Figure 3B). The combined method of microwave assisted acid pre-treatment and neutrase hydrolysis greatly increased the hydrolysis efficiency. The hydrolysate of bone collagen at 1.5 hours achieves the same peptide distributions of traditional enzymatic hydrolysis at 3 hours (Figure 3).

### 2.3. Effect of Microwave Assisted Acid Pre-treatment on the Antioxidant Activity

**Figure 4.**
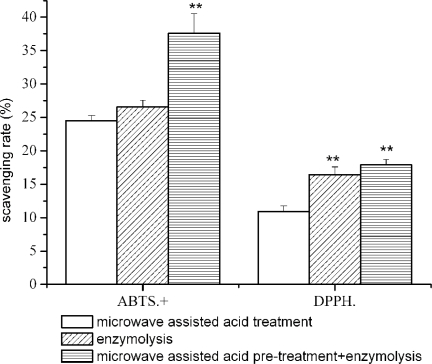
Scavenging activity of bone collagen hydrolysate on DPPH radical and ABTS. Results are presented as the means (n = 3) ± SD. ** Indicates significant difference at (*P* < 0.01).

Figure 4 shows the DPPH and ABTS radical scavenging ability of three bone collagen hydrolysates. A combination of microwave assisted acid pre-treatment and neutrase hydrolysis (for 3 h) lead to the higher ABTS radical scavenging activity (37.6 ± 2.9%); whereas, the hydrolysate of neutrase treatment has similar DPPH scavenging to that of microwave assisted acid pre-treatment plus neutrase treatment (Figure 4).

Collagen is a cheap and resourceful meat byproduct. Potential antioxidative peptides have been identified in fish skin gelatin [24,25]. The antioxidant activity of hydrolysate is dependent on the quantity and amino acid composition of the peptides sequences. Chen *et al*. [26] and Rajapakse *et al.* [27] suggest that antioxidant activity of peptides depended on hydrophobic amino acids. *N*-terminal hydrophobic amino acids, such as Val or Leu give peptide higher antioxidant activity. Hydrophobic amino acids in peptideare are known to tackle lipid free radicals. However, the presence of Pro, Tyr and His in the sequence, could enhance the antioxidant activity of peptides [28–30]. Neutral protease is an exopeptidase that is suitable for producing antioxidant peptides because it cleaves protein at the hydrophobic amino acids site, such as Leu, Phy, Tyr, *etc*. To increase its efficiency, different methods were combined. The collagen structure is majorly made of Gly, Pro and Hydroxyproline. Thus, the combination of microwave assisted acid pre-treatment and enzymatic hydrolysis increases the hydrolysis reaction sites of bone collagen protein and produces more antioxidant peptides.

### 2.4. Effect of Nitrogen Protection on DH and Antioxidation Activity

**Figure 5.**
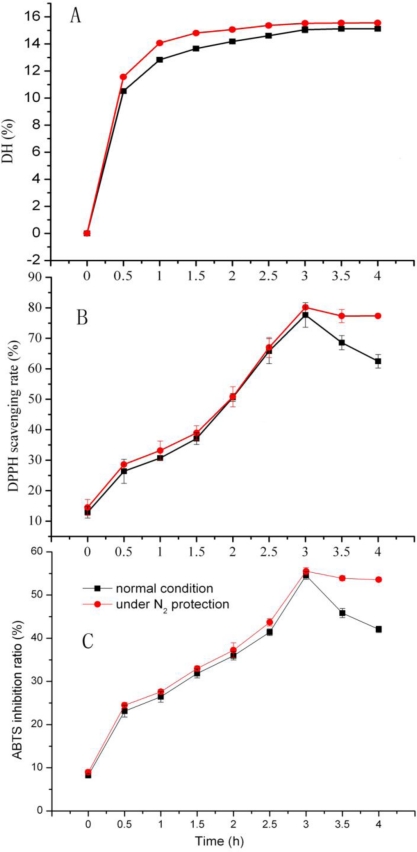
Antioxidant activity and degree of hydrolysis of swine bone collagen hydrolysates by Neutrase under nitrogen protection. Results are presented as the means (n = 3) ± SD. (**A**) is significantly different at (*P* < 0.01); (**B**) and (**C**) are significantly different at (*P* ≤ 0.05).

We found that when the reaction is completed, DH does not change, and the antioxidant activity of hydrolysate decreased significantly; however it might be due to antioxidant peptides that react with environmental oxygen. Hydrolysis under inert gas might protect oxygen-sensitive amino acids, such as Pro fromoxidation. During 4 h of enzyme hydrolysis, it was found that DH was always significantly higher (*P* < 0.01), when hydrolysis was carried out under nitrogen protection, than under air (Figure 5). The DPPH and ABTS scavenging rate of hydrolysate increased hydrolysis time up to 3 h, and decreased dramatically thereafter. Moreover, the nitrogen protection helped to maintain the activity of scavenging ability significantly (*P* ≤ 0.05), as shown in Figure 5.

## 3. Experimental

### 3.1. Materials

Swine bone collagen (containing 68.23% protein) was purchased from Luohe Wulong Gelatin Co., Ltd. (Luohe, China). 1,1-diphenyl-2-picrylhydrazyl (DPPH) and 2,2’-Azinobis (3-ehtylbenzothiazoli-6-sulfnicAcid) (ABTS), Acid protease (63200 U/g), were purchased from Sigma Chemical Co. (Shanghai, China). Neutral proteinase from *Bacillus subtilis* strain with activity of 47600 U/g, alcalase from *Bacillus subtilis* with specific activity of 174500 U/mL and papain with 132700 U/g activity were purchased from Sunson Industry Group Co., Ltd. (Beijing, China), Novozymes Co. Ltd. (Beijing, China) and Pangbo Bio-products Co., Ltd. (Nanning, China), respectively.

### 3.2. Preparation of Collagen Peptides

#### 3.2.1. Microwave Assisted Acid Pre-treatment of Bone Collagen

Five grams of bone collagen was treated with hydrochloric acid, sulfuric acid, phosphoric acid or citric acid (2 mol/L, 30 mL), respectively, then exposed to 510 W microwave for 210 s using a microwave oven “NN-S563JF” (Matsushita, Japan).

#### 3.2.2. Protease Hydrolysis of Pre-Treatment of Bone Collagen

In our preliminary study, we estimated the degree of hydrolysis (DH) of collagen using different commercial proteases (neutrase, alcalase, Papain and Acid protease). It was found that neutrase gave higher DH than the other enzymes (unpublished data). Therefore the antioxidant activity was investigated on peptides obtained from neutrase hydrolysis.

Bone collagen of microwave assisted acid pre-treatment was adjusted to adapted pH value using NaOH, mixed with distilled water, then homogenized for about 2 min. Protease was added to the mixture for hydrolysis process at a suitable temperature for the scheduled time. At the end of the hydrolysis period, the mixtures were heated in boiling water for 10 min to inactivate the protease. The hydrolysates were cooled then centrifuged for 15 min at 5,000 rpm using centrifuge 5804 R, Oppendorf AG 22331 (Hamburg, Germany), the supernatants were lyophilized and stored in adesiccator until further use.

### 3.3. Determination of Molecular Weight Distribution (MW)

The MW distribution was estimated using a Waters 600 Series HPLC (Water 600, Milford, MA) with a TSKgel 2000 SWXL (300 mm × 7.8 mm) and a UV detector (Waters 2487, Milford, MA) working at 220 nm. The mobile phase consisted of acetonitrile/water/trifluoroacetic acid (45:55:0.1, v/v/v) at a flow rate of 1.0 mL/min. Cytochrome C (MW12500), *Bacillus* enzyme (MW1450), Gly-Gly-Tyr-Arg (MW451) and Gly-Gly-Gly (MW189) were used as the MW markers.

### 3.4. DPPH Radicals Scavenging Activity Assay

The scavenging effect of swine bone collagen on 1,1-diphenyl-2-picrylhydrazyl (DPPH) free radical was measured according to the method of Shimada *et al.* [31] with little modification. Two milliliters of sample solution (ratio 1:1 v/v sample/ethanol) were added to 2 mL of 0.1 mM DPPH dissolved in 95% ethanol. The mixture was shaken and left for 30 min at room temperature, and the absorbance of resulting solution was read at 517 nm. A lower absorbance represents a higher DPPH scavenging activity. The scavenging effect was expressed as shown in the following equation:

(1) $$\text{DPPH scavenging activity }(\%)\;=\;\frac{\left(\text{Blank absorbance }-\text{ Sample}\;\text{absorbance}\right)}{\text{Blank absorbance}}\times \;100$$

### 3.5. ABTS• Scavenging Activity Assay

ABTS radical scavenging activities of swine bone collagen were determined by the method described by Re *et al.* [32], with slight modification. A stock solution of ABTS radicals was prepared by mixing 5.0 mL of 7 mM ABTS solution with 88 μL of 140 mM potassium persulfate, and kept in the dark at room temperature for 12–16 hours. An aliquot of stock solution was diluted with Phosphate buffer, PB (5 mM, pH 7.4) containing 0.15 M NaCl, in order to prepare the working solution of ABTS radicals to an absorbance of 0.70 ± 0.02 at 734 nm. Swine bone collagen peptides were mixed 1:4 (v/v) with diluted ABTS, or only buffer (for the control), incubated for 10 min at room temperature in the dark, and then absorbance was measured at 734 nm. The percent reduction of ABTS^+^ to ABTS was calculated according to the following equation:

(2) $$\text{ABTS }(\%)\;=\;\left(1-\frac{\text{Absorbance of sample}}{\text{Absorbance of control}}\right)\times 100$$

### 3.6. Statistical Analysis

Each experiment was performed in three determinations. All antioxidant assays were carried out in triplicate. Results were subjected to analysis of variance using the SAS (SAS Institute, Inc., Cary, NC, U.S.), and significant differences (*P* < 0.05) or (*P* < 0.01) between individual means were identified by the least significant difference (LSD) procedure. Data were presented as means and standard deviations.

## 4. Conclusion

This research showed that the swine bone collagen hydrolysate has high antioxidant activity under the optimum conditions of neutrase hydrolysis. Microwave assisted acid pre-treatment with nitrogen protection helped also to maintain the activity of scavenging ability. The molecular weight distributions of peptide from the microwave assisted acid pre-treatment and neutrase hydrolysis resulted in low molecular weight, mainly <1000 Da. However, the lower molecular weight was found to be strongly correlated with its antioxidant activity. Microwave assisted acid pre-treatment and nitrogen protection hydrolysis could be a quick method to produce antioxidative peptides from swine bone collagen.

## Figures and Tables

**Table 1.** Comparison of acids used for microwave-assisted hydrolysis.

	**Hydrochloric Acid**	**Sulfuric Acid**	**Phosphoric Acid**	**Citric Acid**
DH (%)	5.22 ± 0.18	4.80 ± 0.15	6.17 ± 0.08	4.16 ± 0.21
ABTS+ (%)	6.5 ± 0.23	4.8 ± 1.02	28.0 ± 0.11	21.2 ± 0.12
DPPH·(%)	9.2 ± 0.17	12.1 ± 0.09	9.9 ± 0.09	10.1 ± 0.10
